# Integrating implementation science in clinical research to maximize public health impact: a call for the reporting and alignment of implementation strategy use with implementation outcomes in clinical research

**DOI:** 10.1186/s13012-020-01060-5

**Published:** 2020-11-25

**Authors:** Brittany N. Rudd, Molly Davis, Rinad S. Beidas

**Affiliations:** 1 Institute for Juvenile Research, Department of Psychiatry, University of Illinois at Chicago, 1747 West Roosevelt Road, Chicago, IL 60608 USA; 2 Department of Psychiatry, Perelman School of Medicine, University of Pennsylvania, Philadelphia, PA USA; 3 Penn Implementation Science Center, Leonard Davis Institute (PISCE@LDI), Philadelphia, PA USA; 4 Department of Medical Ethics and Health Policy, Perelman School of Medicine, University of Pennsylvania, Philadelphia, PA USA; 5 Department of Medicine, Perelman School of Medicine, University of Pennsylvania, Philadelphia, PA USA

**Keywords:** Reporting guidelines, Implementation strategy specification, Translational science, Public health

## Abstract

**Background:**

Although comprehensive reporting guidelines for implementation strategy use within implementation research exist, they are rarely used by clinical (i.e., efficacy and effectiveness) researchers. In this debate, we argue that the lack of comprehensive reporting of implementation strategy use and alignment of those strategies with implementation outcomes within clinical research is a missed opportunity to efficiently narrow research-to-practice gaps.

**Main body:**

We review ways that comprehensively specifying implementation strategy use can advance science, including enhancing replicability of clinical trials and reducing the time from clinical research to public health impact. We then propose that revisions to frequently used reporting guidelines in clinical research (e.g., CONSORT, TIDieR) are needed, review current methods for reporting implementation strategy use (e.g., utilizing StaRI), provide pragmatic suggestions on how to both prospectively and retrospectively specify implementation strategy use and align these strategies with implementation outcomes within clinical research, and offer a case study of using these methods.

**Conclusions:**

The approaches recommended in this article will not only contribute to shared knowledge and language among clinical and implementation researchers but also facilitate the replication of efficacy and effectiveness research. Ultimately, we hope to accelerate translation from clinical to implementation research in order to expedite improvements in public health.

**Supplementary Information:**

The online version contains supplementary material available at 10.1186/s13012-020-01060-5.

Contributions to the literature

- Clinical researchers rarely specify all of the implementation strategies used within clinical trials or align those strategies with measured implementation outcomes which stymies translation from clinical to implementation research.
- We propose that revisions to frequently used reporting guidelines in clinical research (e.g., CONSORT, TIDieR) are needed to nudge and support clinical researchers in this reporting.
- We review current guidelines for specifying implementation strategies (e.g., StaRI).
- We offer pragmatic methods for specifying implementation strategy use in clinical research, provide a case study, and advocate for interdisciplinary collaboration between clinical researchers and implementation scientists to facilitate reporting.
- These approaches can narrow the gap between stages on the translational research spectrum, thereby helping reduce the research-to-practice gap in public health.

## Background

The National Institutes of Health’s National Center for Advancing Translational Sciences promotes a model of translational science with five interrelated stages of research, which create a spectrum, beginning with basic research and culminating in public health impact. As implementation researchers who study methods to promote the systematic uptake of evidence-based clinical practices^1^ (hereafter referred to as clinical interventions) into routine care to improve health [1], we are the final frontier to ensure public health impact [2]. However, our research draws upon the findings of our colleagues who work in earlier stages of the translational spectrum, particularly clinical researchers who study the efficacy and effectiveness of clinical interventions. Given the clear synergy between clinical and implementation research, many research teams have called for the integration of implementation science earlier on the translational spectrum [3–6]. In the current debate, we propose a novel way to harness this synergy—revising frequently used reporting guidelines in clinical research to support researchers in reporting the implementation strategies they are already using and aligning these strategies with the implementation outcomes they are already measuring (e.g., fidelity) in their clinical trials.

Implementation scientists often look to the results of clinical researchers’ randomized controlled trials (RCTs) to know which clinical interventions are ready for implementation and scale-up. In order to conduct rigorous RCTs, clinical researchers expend significant resources to monitor the clinical intervention’s delivery and support its implementation (i.e., quality-monitoring systems). If the clinical intervention is not delivered with fidelity, the resulting estimate of the intervention’s effect is biased. Thus, within the context of clinical research, including both tightly controlled efficacy trials and pragmatic effectiveness studies deployed across a range of settings (e.g., hospitals, outpatient clinics, schools), investigators often allocate extensive resources to achieve and maintain high levels of fidelity to the clinical intervention. This means many clinical researchers use elements of implementation science in their research programs [7]. However, manuscript submission page limits and differences in terminology used by scientists across the translational research spectrum have left these “elements,” which we would refer to as “implementation strategies” (i.e., methods or techniques used to enhance the adoption, implementation, and sustainability of a clinical intervention [3, 8]) in implementation science, underreported or unlabeled in the clinical research stage of the translational science spectrum.

Comprehensive reporting guidelines for implementation strategies within implementation research exist, including the Standards for Reporting Implementation Studies (StaRI) guidelines [9, 10], which are endorsed by* Implementation Science*. In this debate, we argue that the lack of comprehensive reporting of implementation strategy use within clinical research is a missed opportunity to efficiently narrow the research-to-practice gap. We view this omission as the natural consequence of distinct fields of research progressing on their own, relying on distinct nomenclature and methodologies, rather than in synchrony. To encourage our clinical research colleagues to include information regarding implementation strategies when reporting on their efficacy and effectiveness studies, we provide suggestions for revising the reporting guidelines they frequently use. We offer methods to pragmatically apply and integrate existing taxonomies from implementation research in order to prospectively and retrospectively specify implementation strategy use and align those strategies with measured implementation outcomes within clinical research. Specification of implementation strategies within clinical research may not only facilitate replication of efficacy and effectiveness results but also contribute to shared knowledge and language among clinical and implementation researchers. We believe such efforts will accelerate translation from clinical research to implementation research, which we hope will expedite improvements in public health.

## Reporting guidelines

Standard reporting guidelines, which have become a routine part of manuscript submissions in the past decade and are revised periodically to include advances in science [11–16], serve as a key mechanism for clearly delineating the processes by which a research study is conducted [17]. Inadequate reporting is “avoidable waste” that reduces the usefulness of research [18]. The Consolidated Standards of Reporting Trials (CONSORT 2010, [16]) and Standard Protocol Items: Recommendations for Interventional Trial (SPIRIT 2013, [19]) statements provide comprehensive evidence-based guidelines for the reporting of RCTs and clinical trial protocols, respectively. For both guidelines, there are sections devoted to the specification of the clinical interventions under evaluation including specifying how fidelity, a prominent implementation outcome, was monitored. However, they were primarily designed for biomedical research. These guidelines can also be “extended” to be relevant for new contexts and populations. For instance, CONSORT extension statements were developed for nonpharmacologic treatments such as surgery, rehabilitation, education, psychotherapy (CONSORT NPT, [11]), and social and psychological interventions (CONSORT SPI, [13, 15]).

When reporting a complex intervention, an alternative approach to using guidelines like CONSORT NPT and CONSORT SPI is to use the Template for Intervention Description and Replication (TIDieR, [20]) extension, which is an extension specifically to the intervention sections of CONSORT 2010 and SPIRIT 2013. TIDieR is the most comprehensive guide to specifying clinical interventions, with the overarching stated goal to “prompt authors to describe interventions in sufficient detail to allow their replication.” TIDieR, like CONSORT and SPIRIT, also can be extended. For example, Cotterill and colleagues [21] recently proposed an extension to TIDieR for use outside of clinical trials and in applied research settings.

We are proposing that revisions to TIDieR, as well as CONSORT, SPIRIT, and any other guidelines used to support the reporting of studies that aim to evaluate the efficacy or effectiveness of a clinical intervention, are necessary to specifically elicit implementation strategy use within clinical trials. While TIDieR prompts authors to briefly describe implementation strategies, there is no guidance for comprehensive implementation strategy specification. With such limited prompting, clinical researchers lack guidance on how to specify the full range of implementation strategies they are using in their research, and thus, the default is often to exclude this important information from manuscripts. Revising the defaults to elicit implementation strategy reporting offers an avenue toward enhancing implementation strategy reporting in clinical research [22]. Such revisions to existing reporting checklists may nudge clinical researchers to provide important information on implementation strategies [23], which would provide the opportunity to align those implementation strategies with the implementation outcomes they are already reporting.

The field of implementation science has much to offer with regard to the specification of implementation strategies, which represent the core interventions of our science. Proctor, Powell, and McMillen [8] developed the most comprehensive method for implementation strategy specification, proposing that implementation strategy specification requires three steps: “Name it,” “Define it,” and “Operationalize it” (“it” being the implementation strategy).

### “Name it” and “Define it”

Thought leaders have argued for the use of standard names and definitions, drawn from standardized implementation strategy taxonomies, to support future replication and research synthesis in implementation research [8]. The most widely used implementation strategy taxonomy in the USA was derived from a compiled menu of 68 implementation strategies used to promote health evidence-based practice implementation that were grouped into overarching categories including planning, educating, financing, restructuring, managing quality, and attending to the policy context [24, 25]. This menu was then later refined through expert international consensus to yield standard, agreed upon, names and definitions. This refined taxonomy of 73 implementation strategies is known as the Expert Recommendations for Implementation Change (ERIC) Project and is organized into conceptually distinct categories including (1) engage consumers, (2) use evaluative and interactive strategies, (3) change infrastructure, (4) adapt and tailor to the context, (5) develop stakeholder interrelationships, (6) utilize financial strategies, (7) support clinicians, (8) provide interactive assistance, and (9) train and educate stakeholders [26].

### "Operationalize it"

Proctor and colleagues propose dimensions that constitute the adequate operationalization of implementation strategies. These include (1) specifying the actors (i.e., who delivers the strategy); (2) the actions; (3) the targets of the action (i.e., toward what or whom—also known as the unit of analysis—*and* at what level); (4) temporality (i.e., when or at what phase); (5) dose (i.e., at what frequency and intensity); (6) the implementation outcomes affected; and (7) justification (i.e., based upon what theoretical, empirical, or pragmatic rationale). See Proctor and colleagues [8] for a more comprehensive description of this method including examples of comprehensive implementation strategy specification.

### AACTT

Building on Proctor and colleagues’ [8] work, Presseau and colleagues [27] highlight the importance of aligning well-specified implementation strategies to well-specified implementation outcomes, particularly when measuring adoption and sustainment. Presseau and colleagues [27] note the “centrality of behavior” in implementation science, emphasizing that one of the primary outcomes of implementation strategies is to get “someone… somewhere…to do something…differently” (p. 2). Presseau and colleagues argue that this means that implementation outcomes are made up of several behaviors delivered by multiple stakeholders at different levels of the organization, and that each of these behaviors needs to be defined in terms of who performs it, for/with whom, when, and where. Presseau and his colleagues proposed the Action (discrete behavior), Actor (who does the behavior), Context (physical, emotional, or social setting in which the behavior occurs), Target (individual[s] for, with, or on behalf of whom the action is performed), and Time (time period and duration) (AACTT) framework to improve upon the specification of implementation outcomes, such as those specified in Proctor and colleagues’ [28] evaluation framework (e.g., adoption, sustainability). Presseau and colleagues emphasize that breaking down the actions involved in each implementation outcome may facilitate better planning for and alignment between implementation strategies and outcomes.

## Missed opportunities to move the needle on public health

A recent review of psychological literature found that clinical researchers do not comprehensively report in their manuscripts the information needed to successfully implement interventions [29]. TIDieR’s inclusion of implementation strategies within its checklist represents a step forward with regard to supporting researchers in specifying implementation components generally and implementation strategies specifically within clinical research. However, the guidance for specifying implementation strategies is limited. We propose that comprehensive reporting of implementation strategies within clinical research, and the resulting ability to connect those strategies to implementation outcomes, may accelerate public health impact by facilitating replication, research-to-practice implementation, and designing for implementation.

### Replication

One of the overarching goals of reporting guidelines is to support the future replication of clinical intervention results, and our proposal aligns with recent calls to improve the transparency of clinical research reporting [30]. While guidelines have improved reporting [17], they have not necessarily improved research-to-practice translation. Implementation strategies within efficacy and effectiveness studies directly affect the ways in which clinical interventions are delivered and the effects they have on resulting health outcomes. Thus, if investigators do not precisely report their use of implementation strategies, this hinders replication by other investigators. For instance, if completing an effectiveness study in a medical setting required creating new clinical teams yet that hiring process is never described in a manuscript or reporting checklist, researchers trying to replicate this study may do so without knowledge of the personnel needed to achieve similar effects. The lack of reporting regarding implementation strategy use likely contributes to observed replication failures and the attenuation of effect sizes associated with clinical interventions once delivered in community settings [31].

### Facilitating research-to-practice implementation

A second overarching goal of reporting guidelines is to support research synthesis through systematic reviews and meta-analyses. Improved reporting of implementation strategy use, implementation outcomes, and the alignment of the two within efficacy and effectiveness studies would facilitate the inclusion of this information in future systematic reviews and meta-analyses. Implementation strategy use could be a new category that is coded, alongside other important intervention characteristics, during data extraction for a meta-analytic study. This would then allow for the analysis of associations between the number and types of implementation strategies used and the effect sizes reported across efficacy and effectiveness studies, which might provide important information to facilitate research-to-practice implementation.

Transparent reporting of implementation strategies and alignment with implementation outcomes provide a clear picture for clinicians and administrators regarding what implementing a given clinical intervention entails, thus setting them up for success. Leaders reading the results of the clinical trial can envision who will be involved in the clinical intervention, the sequences of behaviors from the system to provider level, and the implementation strategies that need to be enacted to support each behavior. In addition, if meta-analytic results reveal that for specific contexts, a certain combination of implementation strategies is ideal for achieving desired health outcomes, this may lead future adopters of said clinical intervention to also utilize that combination of implementation strategies. Alternatively, with implementation strategies clearly connected to implementation outcomes, leaders will have a better understanding of which outcomes may suffer if they choose not to enact a strategy. Given that decisions about the implementation of a clinical intervention are often made by leadership and impact the organization as a whole, comprehensive reporting of the implementation strategies used in efficacy and effectiveness trials and their connected outcomes provides leadership with valuable information as they consider which clinical interventions to adopt in their settings.

### Designing for dissemination and implementation

As Lane-Fall, Curran, and Beidas [4] note, at the efficacy stage of research for a clinical intervention, a clinical researcher is unlikely to explicitly study implementation. However, clinical researchers can and should attempt to design the clinical intervention with deployment in mind. This entails thinking through both the clinical intervention and implementation strategies needed to successfully deploy the clinical intervention at scale in the community. By prompting clinical researchers to think through and report the use of implementation strategies earlier in the translational process, including how those strategies relate to implementation outcomes, intervention developers may create interventions that are primed for dissemination, adoption, implementation, and sustainment. Importantly, some researchers already engage in the important work of designing for implementation [32], but the field lacks clear expectations and methods for reporting these efforts in publications as well as in grant proposals. By encouraging clinical researchers to report implementation strategy use and by delineating standard methods for reporting, we provide an opportunity for our clinical research colleagues to share their approaches to navigating and overcoming the many challenges of testing and deploying clinical interventions.

### Accelerating translational science

With the ultimate goal of reducing the time it takes to achieve positive public health changes, researchers have started to develop methods to accelerate the translation from clinical research to implementation research. Most notably, Curran and colleagues [3] specified hybrid effectiveness-implementation research designs, which combine elements of clinical research and implementation research to understand both patient and implementation outcomes in a single study. In a similar vein, we propose that moving toward comprehensive specification of implementation strategy use in clinical research is another way to reduce the time it takes to move from clinical research to public health impact. Specifying implementation strategy use within the clinical research phase of the translational spectrum of science allows for the explicit alignment between strategies and outcomes and will provide implementation researchers with information to tailor implementation strategies to enhance the uptake and sustainability of clinical interventions in community settings.

## Proposed methods for prospectively and retrospectively specifying implementation strategy use in efficacy and effectiveness research

We draw from existing efforts to prospectively and retrospectively track and report implementation strategies within implementation research. Rogal and colleagues [33–35] surveyed implementation practitioners regarding their use of ERIC strategies to promote various health behaviors such as hepatitis C virus medication adherence. Importantly, Rogal and colleagues found that it was feasible to present stakeholders with the list of ERIC strategies and that stakeholders were able to reliably select the ones they used. Rogal and colleagues’ survey quantifies the number and type of implementation strategies used but does not operationalize their use. Bunger and colleagues [36] developed an activity log to capture and operationalize the implementation strategies developed outside of formal meetings and in everyday practice. Team members who were involved in implementation but were non-implementation scientists recorded all activities they viewed as being related to successful implementation (i.e., actions, methods, events, or efforts to promote adoption and implementation of project components) using the activity log by listing each activity they engaged in, including the purpose (to identify the type of strategy), estimated length of time (to estimate dosage), and individuals involved (to specify actors). Bunger and colleagues piloted using this activity log both prospectively and retrospectively. The data collected in the activity logs was coded by implementation experts to specify the implementation strategies used in the implementation trial using Powell and colleagues’ [25] compilation of strategies. The implementation strategies identified were then operationalized using Proctor and colleagues’ [8] guidelines. Boyd and colleagues [37] proposed an alternative method for prospectively assessing and operationalizing the use of implementation strategies within the context of an implementation study. Boyd and colleagues recorded and transcribed the tri-weekly implementation meetings that were held to support implementation and subsequently coded the transcripts for implementation strategy use with Powell and colleagues’ [24] compilation of strategies and Proctor and colleagues’ [8] guidelines for operationalization, similar to Bunger and colleagues [36]. Boyd and colleagues also coded whether the implementation strategy was planned or enacted (i.e., actually used). These methods can support researchers in tracking their use of implementation strategies across the course of a study.

In terms of existing reporting guidelines, the gold-standard option for specifying implementation strategies is the Standards for Reporting Implementation Studies (StaRI) statement [9, 10]. StaRI was designed for reporting implementation research (i.e., when either an implementation strategy or both an implementation strategy and clinical intervention are under investigation). StaRI builds upon TIDieR by prompting investigators to both specify the clinical intervention under investigation using TIDieR guidelines and the implementation strategy under investigation using Proctor and colleagues’ “Name it,” “Define it,” and “Operationalize it” guidelines [27]. Because clinical researchers typically use CONSORT statements and TIDieR, which do not prompt for the reporting of implementation strategies, and they are not in the habit of using StaRI, the default is to omit this information from their manuscripts. Thus, we believe that TIDieR and CONSORT revisions are warranted so that clinical researchers are nudged into providing this crucial information in their manuscripts via the reporting checklists they use [23].

As the field of implementation science has matured, it has become quite clear that organizations often need to deploy many implementation strategies to successfully implement an intervention (e.g., [33, 38]). For example, in Rogal’s [33–35] aforementioned work, between 23 and 27 implementation strategies were used to support the health interventions. Reporting the implementation strategies used is only one component of a multi-item reporting checklist (e.g., item 9 of StaRI’s 27 item checklist). Reporting dozens of strategies within the reporting checklists could become bulky and challenging to follow for the reader. An integrated tool that complements available reporting guidelines could streamline the implementation strategy operationalization process. Additional file 1 provides an example of what we believe to be such a pragmatic tool. Given Rogal et al.’s [33] work that demonstrates that it is feasible to provide the entire list of ERIC strategies to non-implementation scientists and have them reliably select which ones they used, the pragmatic implementation strategy reporting tool combines the ERIC taxonomy [25] with Proctor and colleagues’ [8] guidelines for implementation strategy reporting. Presseau and colleagues’ [27] AACTT model was originally designed to clarify and specify behaviors that makeup implementation outcomes and implementation strategies are similarly clusters of behaviors. Thus, we expand Proctor and colleagues’ model to integrate components of Presseau et al.’s AACTT model where appropriate to allow for further specification of implementation strategies. Ultimately, the current tool allows clinical researchers to easily locate a comprehensive and standardized list of implementation strategies alongside prompts to specify details of each strategy used including the outcomes the strategies aim to improve. The tool thereby streamlines comprehensive implementation strategy reporting, particularly when reporting on more than one implementation strategy.

**Fig. 1:**
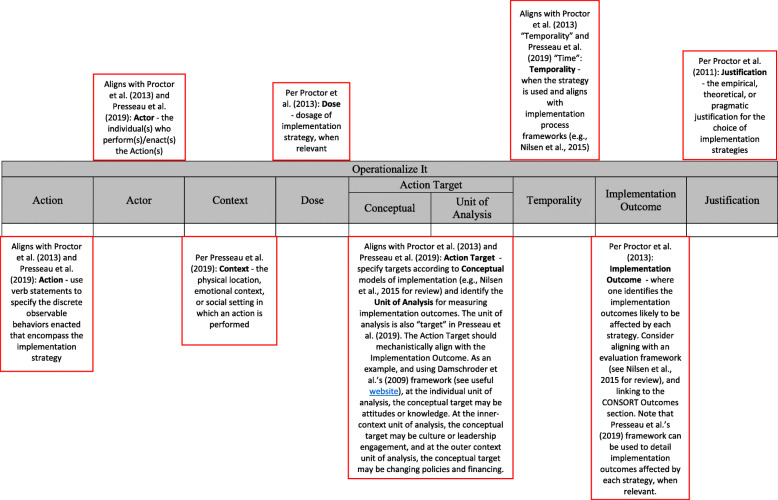
Specification of implementation strategies

In the first column of the tool, the researcher identifies whether the ERIC strategy listed in column B and defined in column C was used. Columns D-L prompt for operationalization of the implementation strategy, as proposed by Proctor, Powell, and colleagues [8] and with additions from Presseau and colleagues [27]. Figure 1 provides a detailed description of each of these items. In specifying the Action Target, we provide an opportunity to both specify the unit of analysis and the conceptual target. In specifying the unit of analysis, it may be helpful to review and select units most commonly used in implementation determinants frameworks [39–41], such as the Consolidated Framework for Implementation Science [39]. Specifying the conceptual target involves clearly delineating the proximal focus of the implementation strategy, which at the individual level may include changing behaviors or attitudes, at the inner-context level may include shifting culture or leadership behavior, and at the outer context/systems level may include changing policies and financing. When specifying temporality, we recommend selecting an implementation process framework [41] to represent the phased nature of implementation. Some determinant frameworks also integrate the process of implementation, such as the Consolidated Framework for Implementation Research [25] and Exploration, Preparation, Implementation, and Sustainment framework [40]. Similarly, to specify implementation outcomes, we recommend referencing an evaluation framework such as Proctor and colleagues’ Outcomes for Implementation Research [28] or the Reach, Effectiveness, Adoption, Implementation, and Maintenance framework [42].

The tool presented is flexible and can be used to advance translational science in several ways. This tool was initially designed with clinical researchers’ needs in mind and with an eye toward integrating with reporting guidelines designed for clinical trials (e.g., CONSORT, TIDIER). However, we also believe that it may be useful within the context of implementation research through integration with guidelines such as StaRI. When this is the case, we encourage implementation researchers to consider using Presseau et al.’s [27] AACTT framework to specify implementation outcomes, particularly when evaluating adoption or sustainment. While the AACTT framework can be used by clinical researchers to detail their implementation outcomes, it was specifically designed for specifying adoption and sustainment and these outcomes typically are not the focus of efficacy or effectiveness of clinical trials. We believe the tool presented can be used in clinical and implementation grant applications to propose implementation strategies and align those strategies with implementation outcomes. It can be used prospectively, during weekly or monthly research team meetings in the planning and active phases of a trial to track implementation strategy use. Then, at the end of the trial, the information can be summarized and described in the manuscript. Alternatively, this information can be gathered retrospectively, using calendars and project files to support recall. This tool was designed to be accessible to researchers without expertise in implementation science, so all of the information needed to adequately specify implementation strategy use is in one place.

## Using the pragmatic implementation strategy reporting tool

We provide an illustrative example of using this tool to specify implementation strategy use with Dorsey and colleagues’ [43] recent effectiveness study of a task-shifted (i.e., the providers did not have specialized training in behavioral health care) version of trauma-focused cognitive behavioral therapy for children who experienced parental death and post-traumatic stress in Kenya and Tanzania. The corresponding author of the trial completed the tool (see Additional file 2). In addition, two authors of this manuscript (BNR and MD) collaboratively coded the published manuscript for mention of implementation strategy use. Table 1 compares the results of the author completed tool to what was reported in the text and highlights how using such a tool can communicate additional information about strategy use and alignment with implementation outcomes. For example, the implementation strategies not mentioned in text were frequently those that fell in the preparation phase. These were strategies such as “conducting local needs assessment” and “develop resource sharing agreements,” which researchers often do to optimize the fit of the intervention to the setting, also known as “designing with implementation in mind”^—^important and challenging work that should be highlighted.

**Table 1:** Comparison of implementation strategy reporting by method

Name it: ERIC implementation strategy	Operationalize it								
	Action	Actor	Context	Dose	Action target		Temporality	Implementation outcome	Justification
					Conceptual	Unit of analysis			
Use evaluative and iterative strategies									
Conduct local needs assessment	A	A	A	A	A	A	A	A	A
Develop and implement tools for quality monitoring	A	A	A	A	A	A	A	A	A
Develop and organize quality monitoring systems	A	A	A	A	A	A	A	A	A
Stage implementation scale-up	A	A	A		A	A	A	A	A
Provide interactive assistance									
Provide clinical supervision	A, T	A, T	A, T	A, T	A	A, T	A, T	A, T	A
Adapt and tailor to context									
Promote adaptability	A	A	A		A	A	A	A	A
Develop stakeholder interrelationships									
Build a coalition	A	A	A	A	A	A	A	A	A
Identify early adopters	A	A	A	A	A	A	A	A	A
Organize clinician implementation team meetings	A	A	A	A	A	A	A	A	A
Train and educate stakeholders									
Conduct educational outreach visits	A, T	A, T	A, T	A, T	A	A, T	A, T	A, T	A
Conduct ongoing training	A, T	A, T	A, T	A, T	A	A, T	A, T	A	A
Create a learning collaborative	A	A	A	A	A	A	A	A	A
Develop educational materials	A	A	A	A	A	A	A	A	A
Distribute educational materials	A	A	A		A	A	A	A	A
Make training dynamic	A	A	A	A	A	A	A	A	A
Provide ongoing consultation	A, T	A, T	A	A, T	A	A, T	A, T	A	A
Use train-the-trainer strategies	A, T	A, T	A, T	A, T	A	A, T	A, T	A	A
Support clinicians									
Create new clinical teams	A, T	A, T	A, T		A, T	A, T	A, T	A	A
Develop resource sharing agreements	A	A	A		A	A	A	A	A
Engage consumers									
Utilize financial strategies									
Change infrastructure									
Change service sites	A	A	A		A	A	A	A	A

*A* reported by author, *T* coded in text

We believe that comprehensive specification of implementation strategy use in clinical research will ultimately accelerate the translation of research to public health impact. We believe that part of this will be due to enhanced research synthesis in future systematic reviews and meta-analyses. Moreover, decades of efficacy and effectiveness research exist to inform implementation research and, ultimately, public health. Thus, in an effort to expedite the progression from clinical to implementation research, we argue for clinical researchers to report on their use of implementation strategies in their research. We offer a pragmatic tool based on the work of leading implementation scientists that can be integrated with currently used clinical and implementation research reporting guidelines so that we can learn about implementation from existing efficacy and effectiveness studies. We are in the process of piloting this method of (1) coding manuscripts in meta-analyses for their use of implementation strategies using the tool in Additional file 1; (2) asking corresponding authors to complete the tool regarding their clinical trial; and (3) triangulating across our coding and the reports from authors (similar to the example provided above). We are hopeful that the method we are piloting will be incorporated into efficacy and effectiveness research going forward to advance the translation of clinical research to public health impact.

### Alternative viewpoints

#### Do we really need new ways of specifying strategies given existing reporting checklists and other tools?

We carefully considered whether promoting the use of existing tools in their current forms would be sufficient for increasing clinical researchers’ reporting of implementation strategy use. We recognize the merits of resources such as StaRI and approaches utilized by others to track and specify implementation strategy use [35–37]. Our goal was to avoid duplicating efforts while also providing a pragmatic way for researchers to report their implementation strategies as well as align those strategies to their implementation targets and outcomes. Currently, despite the availability of StaRI, implementation strategies used within intervention studies are not reported in a systematic way. Thus, we elected to propose revising the reporting guidelines clinical researchers often use so that these researchers can highlight the implementation strategies that allowed their intervention trials to be carried out. We eagerly invite these and other viewpoints to be expanded upon in future manuscripts to further this discussion and advance implementation strategy reporting across disciplines.

#### Is it fair to expect clinical researchers to report on implementation strategies?

Some may argue that researchers should stick to their areas of expertise and that expecting researchers to obtain and apply knowledge from other fields is unnecessary. We propose that clinical researchers do not need a deep understanding of implementation science to accurately and adequately report on the implementation strategies used in their intervention trials. However, we believe that without shared knowledge and language across scientific fields, many key implementation strategies that are pertinent to delivering an intervention and thereby advancing public health are left undescribed and uncaptured for future replication. This has the potential to further widen the very research-to-practice gap that implementation scientists seek to dissolve.

While a deep understanding of implementation science is not imperative for clinical researchers to be able to use the pragmatic reporting methods described above, we do advocate for all researchers to receive training across the translational research spectrum whenever possible. In order to provide a foundational understanding of implementation science that ultimately facilitates transparent implementation strategy reporting, just as clinical research programs require basic science classes, we believe that they should require introductory courses in implementation science. For clinical researchers interested in implementation science training now, the Society for Implementation Research Collaboration keeps an up-to-date list of training opportunities (find it here https://societyforimplementationresearchcollaboration.org/dissemination-and-implementation-training-opportunities). As clinical researchers receive more exposure to implementation science and use research designs that integrate elements of clinical and implementation research, we may observe an increase in clinical researchers with this training or who include implementation scientists on their team. Leveraging opportunities for collaboration between clinical researchers and implementation scientists will be beneficial for designing and reporting implementation strategies as well as for accelerating translational science. Until then, aligned with efforts to make implementation science accessible [44], it is our view that we must make reporting of implementation strategies accessible to clinical researchers by revising the guidelines they already use.

## Conclusions

In this article, we call for clinical researchers to comprehensively report their use of implementation strategies, propose that revisions to the reporting guidelines most frequently used by clinical researchers are needed, offer a pragmatic way for researchers to track and report their use of implementation strategies, and provide an illustrative case study of using these methods. We hope to stimulate discussion in the field regarding specifying implementation strategy use earlier in the translational spectrum of science. We recognize that specifying implementation strategy use will likely increase the length of manuscripts. There is always a tension between thorough clinical trial reporting and journal page limits. However, as journals increasingly move toward open access and hybrid print and online journals, a tool such as the one described here could be included in supplementary material as well as in intervention manuals. We invite readers to develop other innovative and pragmatic methods to support reporting of implementation strategy use in clinical research such as in protocol papers. It is also our hope that the current manuscript will stimulate fruitful discussions between clinical and implementation researchers on ways to bridge the gap between these fields. We believe that these combined efforts will serve to accelerate knowledge translation from clinical research to meaningful improvements in public health.

## Supplementary Information

**Additional file 1.** Pragmatic Implementation Strategy Reporting Tool. Additional File 1 is an integrated tool that complements available reporting guidelines and streamlines the implementation strategy operationalization process.

**Additional file 2.** Operationalization of Implementation Strategies Used in: “Effectiveness of Task-Shifted Trauma-Focused Cognitive Behavioral Therapy for Children Who Experienced Parental Death and Posttraumatic Stress in Kenya and Tanzania: A Randomized Clinical Trial"

## Data Availability

Not applicable.

## Abbreviations

RCT: Randomized controlled trial
StaRI: Standards for Reporting Implementation Studies
CONSORT: Consolidated Standards of Reporting Trials
SPIRIT: Standard Protocol Items: Recommendations for Interventional Trial
NPT: Nonpharmacologic treatments
SPI: Social and psychological interventions
TIDieR: Template for Intervention Description and Replication
ERIC: Expert Recommendations for Implementation Change
AACTT: Action, Actor, Context, Target, and Time

## Footnotes

1 We highlight clinical practices for parsimony, but this debate is relevant to all interventions provided in community settings, including health, educational, and justice settings.
